# Applications in Which Aptamers Are Needed or Wanted in Diagnostics and Therapeutics

**DOI:** 10.3390/ph15060693

**Published:** 2022-06-01

**Authors:** John G. Bruno

**Affiliations:** Nanohmics Inc., 6201 E. Oltorf Street, Suite 400, Austin, TX 78640, USA; jbruno@nanohmics.com

**Keywords:** aptamer, *Cyclospora*, diagnostic, liver fluke, humanized, monoclonal antibody, reproducibility, SELEX, therapeutic, toxic

## Abstract

One strategy for bringing aptamers more into the mainstream of biomedical diagnostics and therapeutics is to exploit niche applications where aptamers are truly needed or wanted for their innate differences versus antibodies. This brief review article highlights some of those relatively rare applications in which aptamers are necessary or better suited to the user requirements than antibodies with explanations for why the aptamer is a necessary or superior choice. These situations include when no commercial antibody exists, when antibodies are excessively difficult to develop against a particular target because the target is highly toxic to host animals, when antibodies fail to discriminate closely related targets, when a smaller size is preferable to penetrate a tissue, when humanized monoclonal antibodies are too expensive and when the target is rapidly evolving or mutating. Examples of each are provided to illustrate these points.

## 1. Introduction

Unfortunately, more than three decades after Tuerk and Gold [1] and Ellington and Szostak [2] first published aptamer selection technology via the systematic evolution of ligands by exponential enrichment (SELEX) method, aptamers are still struggling to be adopted as mainstream diagnostic reagents and therapeutics. In part, this struggle is due to the incumbent and entrenched nature of antibodies. When a reagent works as well as polyclonal or monoclonal antibodies have for many years, it is difficult to supplant such reagents unless the new reagent class has some truly significant advantages, or no antibodies exist for the given target or application.

Admittedly, aptamers have limitations in the diversity of their monomer components (only four natural nucleotides in DNA or RNA versus the 20 natural amino acids in antibodies), potential nuclease degradation in some matrices and potential cross-reactivity issues with more complex target analytes versus comparable antibodies. Thus, rather than trying to compete directly head-to-head against antibodies, which has not worked well thus far for various diagnostic and therapeutic applications, finding niches in which aptamers are needed and wanted seems to be a prudent alternative strategy. Taking advantage of some of the innate differences between aptamers and antibodies, including aptamer nucleic acid composition, smaller size versus antibodies, ability to hybridize and dehybridize as nucleic acids, simple in vitro selection methodology which obviates the use of host animals, and facile site-specific functionalization during chemical synthesis of DNA or RNA, can assist in finding niche applications for aptamers in diagnostics and therapeutics. One can think of a few general situations in which aptamers would be preferable to antibodies such as when lot-to-lot reproducibility is critical or the immune system tolerates an antigen [3,4]. However, more specifically, the author has defined some niche applications listed below with associated examples.

## 2. Diagnostic Applications in Which Aptamers Are Needed or Desired

### 2.1. When No Commercial Antibodies Exist or Are Very Difficult to Obtain

**Figure 1:**
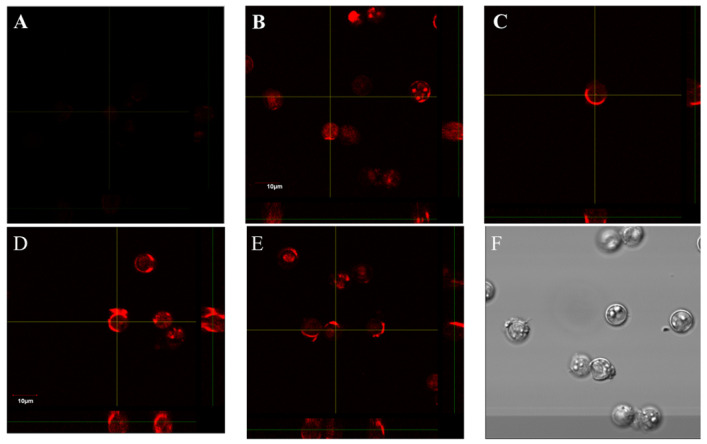
Confocal fluorescence microscopy of *Cyclospora cayetanensis* oocysts after aptamer-based staining. Oocysts were stained with the top 8 DNA aptamer sequences containing 5′-biotin linkers at ~150 µg/mL in phosphate-buffered saline (PBS) for 30 min at room temperature and then washed by centrifugation at 13,000× *g*, and then the pelleted oocysts were resuspended in streptavidin–Texas Red conjugate for 15 min and washed again prior to confocal microscopy. Note that both the oocyst cell surface and interior structures (developing or developed spores) stained with each of the aptamers in panels (**B**–**E**) but not with a scrambled sequence DNA aptamer control shown in panel (**A**). Panel (**F**) shows the appearance of the unstained 8–10 µm oocysts under phase contrast microscopy. Total magnification = 400×.

There is nothing quite so frustrating for a researcher than to need an antibody for a given target antigen, then search Linscott’s directory online to find that no such antibodies are commercially available. This places the researcher in the position of trying to find academic sources or producing their own antibodies which is time consuming and can be expensive. One such example is trying to find antibodies that bind oocysts of the foodborne parasite *Cyclospora cayetanensis.* This emerging parasitic pathogen is becoming a threat to free agricultural trade between Central America, Mexico and the US with annual outbreaks increasing each year [5,6,7,8]. Detection of this parasite on fresh produce and in soils and large volumes of irrigation water is truly a “needle in a haystack” problem; thus, high affinity receptors (i.e., antibodies or aptamers) are required to capture and perhaps concentrate the oocysts. However, Linscott’s directory shows no hits for antibodies against *Cyclospora*, and the lack of commercial antibodies is acknowledged in the literature [9]. Additionally, in private discussions with academic researchers who have tried to develop antibodies against *Cyclospora* oocysts, the author has been informed that it is a very difficult task, and the resulting antibody affinities have been poor. Therefore, the author set out to develop aptamers against *C. cayetanensis* oocysts by the whole cell SELEX method. Unfortunately, because humans are the only animal reservoir for *Cyclospora*, it is extremely difficult to obtain the oocysts in sufficient quantities for several rounds of SELEX. Fortunately, Michael Arrowood at the Centers for Disease Control (CDC) in Atlanta, GA, was able to provide some *C. cayetanensis* oocysts for testing as shown in Figure 1, but to develop the aptamers prior to obtaining the oocysts, the author had to develop aptamers against recombinant proteins (TA4-like antigen and Wall Protein-2) from the oocysts as defined in one of Arrowood’s publications [10]. This appears to have led to aptamers that bind both internally on the developing and developed spores and the exterior cell wall of oocysts as shown in the various panels of Figure 1. The DNA sequences of these *C. cayetanensis* aptamers must remain proprietary and cannot be divulged at present due to the fact of their potential commercial value.

**Figure 2:**
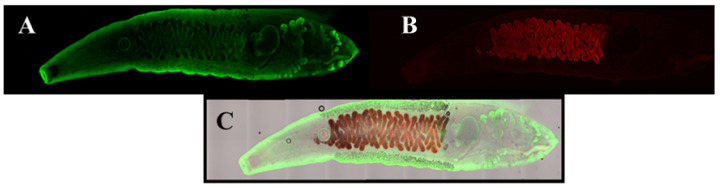
Confocal fluorescence microscopy of anti-Cs44 aptamer-based fluorescence staining of adult *Clonochis sinensis* liver flukes in PBS using the final SELEX round 10 polyclonal aptamers and a method similar to that described in the Figure 1 legend, except that a fluoresceinated streptavidin conjugate was used to detect the 5′-biotinylated aptamer pool on the adult parasites’ surfaces in Panels (**A**,**C**). Panel (**B**) represents red autofluorescence of internal organs but no staining from an aptamer deletion control on the parasite’s surface. Panel (**C**) shows an image of combined fluorescence and brightfield confocal microscopy. Total magnification = 200×.

**Figure 3:**
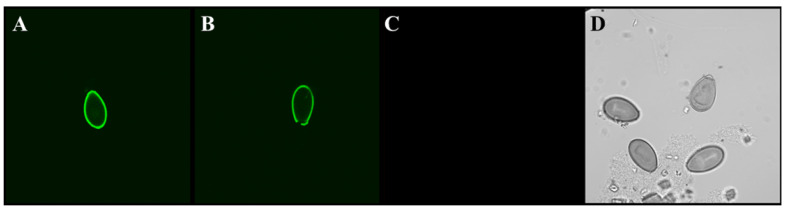
Confocal fluorescence microscopy of *C. sinensis* eggs stained by the same method described in Figure 2, except that the final 5′-biotinylated aptamer pool was raised against the recombinant *C. sinensis* egg protein. Panels (**A**,**B**) show egg surface staining, panel (**C**) represents the appearance of a negative aptamer deletion control and panel (**D**) illustrates the appearance of unstained eggs under phase-contrast microscopy. Total magnification = 400×.

**Figure 4:**
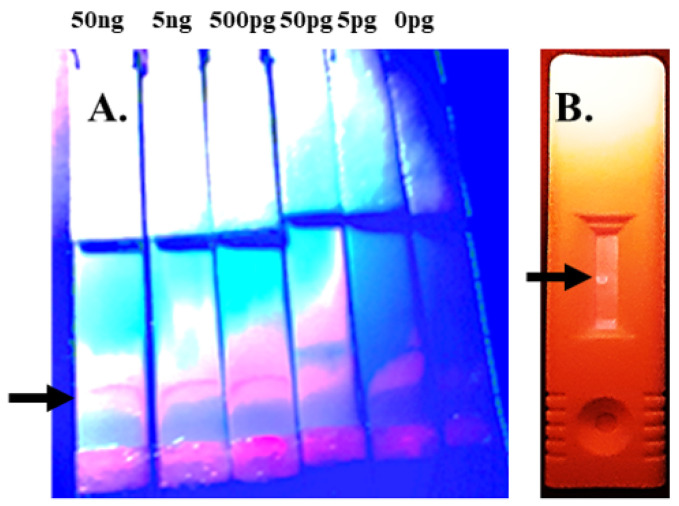
Panel (**A**) illustrates the appearance of a *Clonorchis* Cs44 titration on lateral flow test strips using an aptamer–biotin–streptavidin–red Qdot 655 conjugate that migrated out of the conjugate pads near the bottom of the photograph to bind dried recombinant Cs44 protein lines at the levels indicated above the panel (i.e., 50 ng to 5 pg) with a detection limit of at least 5 pg versus the blank control as indicated by the capture lines shown at the arrow level. Panel (**B**) shows a similar preliminary capture test for 50 ng of the *C. sinensis* recombinant egg protein dried as a dot on the analytical membrane after interaction with an aptamer–biotin–streptavidin–Qdot 655 conjugate to provide proof of concept for eventual lateral flow test strips to detect liver flukes from human serum or diluted fecal suspensions.

Another category of parasites for which there appear to be no commercially available antibodies are liver flukes, which are important, because they have been shown to induce cholangiocarcinoma via chronic inflammation of the bile ducts [11]. The liver flukes, *Clonorchis sinensis* and *Opisthorchis viverrini*, affect tens of millions of people worldwide with by far the greatest concentration in Asia [12]. Rapid microscopic detection of the fluke’s eggs in human fecal smears is challenging. The use of fluorescent probes, such as antibodies or aptamers, can greatly enhance the ability to detect liver flukes in fecal smears under a fluorescence microscope. As shown in Figure 2 and Figure 3, the author’s team developed aptamers capable of detecting both adult *C. sinensis* parasites and their eggs, which may be quite valuable as diagnostics to identify patients with active liver fluke infections in need of treatment to prevent bile duct cancer, especially in Asia. In addition, in negative control experiments, these same aptamer DNA sequences did not bind the sheep liver fluke *Fasciola hepatica* (data not shown), thus proving relative specificity. Because fluorescence microscopy requires some skill and is not accessible to everyone, the author also evaluated the potential for use of the *C. sinensis* aptamers against the recombinant adult surface protein Cs44 from Bioclone Inc. (San Diego, CA, USA) in a lateral flow test strip format as shown in Figure 4. Cs44 might be detectable in human serum during active fluke infections. The end result of those test strip experiments was that red quantum dot (streptavidin-coated Qdot 655 from Invitrogen) plus biotinylated aptamer conjugates prepared according to the author’s published protocols [13,14] detected down to ~5 pg of recombinant Cs44 protein in phosphate-buffered saline as seen in Figure 4A. The analogous aptamer-Qdot lateral flow test strip experiment for *C. sinensis* egg detection produced a 50 ng detection limit for recombinant *C. sinensis* egg protein from Bioclone Inc. as shown in Figure 4B. Again, the DNA sequences of these *C. sinensis* aptamers must remain proprietary due to the nature of their potential commercial value.

### 2.2. When Antibodies Fail to Distinguish Closely Related Variant Targets

There are situations in which antibodies fail to discriminate closely related targets, especially when the target is a small molecule, but aptamers are sometimes able to discriminate these related targets. The classic example is the RNA aptamer developed by Jenison et al. and published in 1994 for discrimination of theophylline from caffeine [15]. The bronchodilator theophylline differs from caffeine by a single methyl group and immunoassays cannot distinguish these targets very well. However, the aptamer developed by Jenison et al. demonstrated a 10,000-fold greater preference for theophylline versus caffeine.

In research performed by the author for the World Anti-Doping Agency (WADA), a series of DNA aptamers were developed that were capable of discriminating the virtually identical natural pituitary human growth hormone (hGH) from recombinant hGH produced in *E. coli* bacteria, which is quite difficult with antibodies [16]. This discrimination was only possible because up to 2% of the recombinant hGH proteins are altered by the bacterial host as proven by mass spectrometry by Hepner et al. [17]. Similarly, the author was able to discriminate isoleucine (I) and threonine (T) variants of prostate-specific antigen (PSA) at position 179, which current antibodies cannot discriminate, using a diaminopurine (DAP)-modified aptamer and three-dimensional information gleaned from a molecular docking model [18,19]. The DAP-modified aptamer yielded an approximately 20% difference in colorimetric absorbance signal in an ELISA-like assay between the I- and T-PSA variants [18].

The new ability to generate at least static rigid three-dimensional models of aptamer-ligand binding [19] is quite valuable, because unlike protein-based antibodies, it is quite facile to inset exotic unnatural bases into aptamers during DNA or RNA synthesis. Thus, with a newfound understanding of the theoretical aptamer binding pocket’s geometry and docking with various targets of interest using free internet PatchDock software, one can modify aptamer binding. These 3D molecular fit knowledge and aptamer modification strategies can lead to better affinity and specificity and thereby better differentiation among variants versus most natural unmodified antibodies which are more difficult to modify.

### 2.3. When Smaller Size Matters

One obvious, yet seldom discussed, aspect of aptamers versus antibodies is their smaller size and weight versus the common IgG antibodies. Average 70–200 base length aptamers generally weigh approximately 20–60 kD as compared to greater than 150 kD IgG antibodies, and the size of the well-known thrombin aptamer is approximately 21 × 25 Å versus the much larger IgG at 122 × 139 Å [20]. For therapeutic applications, smaller size can be detrimental because molecules smaller than 50 kD are rapidly cleared by the kidneys leading to poor pharmacokinetics, but in some diagnostic applications, smaller size can be advantageous. For example, Gomes deCastro et al. [21] discovered that RNA aptamer-based fluorescence staining and microscopy was superior to that of comparable antibody-based immunofluorescence staining, presumably because the much smaller aptamers penetrated to antigens deep in tissues better than the larger antibodies and led to denser epitope staining and greater image intensity and resolution.

Perhaps a less clear example of smaller aptamer size being advantageous versus antibodies may exist in the world of Raman spectroscopy, where both aptamers and antibodies have been conjugated to nanoparticles and used in various detection schemes [22,23,24,25,26,27,28]. For surface-enhanced Raman spectroscopy (SERS), signal intensity is distance-dependent and limited to ~30–60 nm above the surface [29,30,31]. Thus, the 25 Å or 2.5 nm aptamers may enable greater SERS signal intensity by binding analytes closer to the surface (within the useful electrical field) than the larger 139 Å (13.9 nm) IgG plus the often much larger (tens of nm) nanoparticles conjugated to the receptors. Other authors have designed ingenious and very sensitive SERS detection schemes involving Cy3-labeled aptamer release or hybridization probes as well as 15 or 35 nm gold nanoparticles enabling “hot spots” between the nanoparticles that change (decrease) SERS signal intensity at a specific wavenumber (e.g., 1203 cm^−1^) upon binding their cognate targets [26,32,33]. Moreover, the nature of antibodies does not involve hybridization or dehybridization of polymer strands, therefore giving aptamers another unique detection modality that may be advantageous versus antibodies.

## 3. Therapeutic Applications in Which Aptamers Are Needed or Desired

### 3.1. When the Target Is Too Toxic or Lethal for a Host Animal to Develop Antibodies

Although a number of effective snake and spider antivenoms exist that are based on equine antisera, the generation of such antivenoms is risky and precarious for the host animal’s health. One approach to ameliorating the toxicity to host animals is to chemically or physically convert the toxins to more innocuous toxoids, but this process can change the molecular structure of the venom to the point that the resulting anti-toxoid is useless for inhibiting the natural venom. Thus, it is preferable to keep the target venom or toxin in its native three-dimensional conformation and simply develop an aptamer to bind and inhibit the venom in vitro without the need for a host animal at all, such as what the SELEX aptamer development does by obviating the need for host animals. The author and several others have had partial success with neutralizing venom degradative enzymatic activity using specifically developed aptamers [34,35,36]. The use of antivenom aptamers would be additionally advantageous to avoid serum sickness or anaphylaxis upon subsequent venomous bites, although some cytosine-phosphate-guanine (CpG)-centered sequences in aptamers are known to activate Toll-like receptors and lead to inflammation [37].

### 3.2. For Passive Immunity versus the More Expensive Humanized Monoclonal Antibodies

The more common example of aptamer generation to avoid potential damage to host animals is probably generation of aptamers against deadly bacteria or viruses that might kill the host animal. Thus, to date, a number of aptamers have been developed and sequenced for their ability to bind Ebola [38], pandemic influenza strains and other deadly viruses [39,40,41,42,43,44,45,46,47,48,49,50,51,52,53,54,55,56,57,58,59,60,61,62,63,64,65,66,67,68]. Of course, aptamers can work on the same principle of passive immunity that immune sera and humanized monoclonal antibodies do, but at a much-reduced cost and development time. We have all recently witnessed and marveled at the efficacy of the anti-SARS-CoV-2 humanized monoclonal antibody treatments for COVID-19 developed by Eli Lilly and Regeneron. However, comparably effective virus inhibitory results might be obtained by much less expensive, faster and easier development of nucleic acid aptamers against the SARS-CoV-2 receptor binding-domain on the S protein head [68,69]. The same is probably true for aptamers capable of binding Ebola and other hemorrhagic fever viruses [38,70] versus ZMapp’s recombinant tobacco plant-produced antibodies against the filiform Ebola viruses. In addition, if the target is rapidly evolving and mutating, as we have seen with SARS-CoV-2 moving through many variants including delta, mu, omicron, etc., for the last couple of years, aptamers could be developed quickly to bind and neutralize each new variant much more rapidly and with far less expense than comparable humanized monoclonal antibodies. Moreover, if the original or newly emerging pathogen variants are too lethal to work with manually, the SELEX process could be fully automated and hermetically sealed to keep it away from humans altogether. Indeed, such robotic SELEX systems have already been constructed and used to produce aptamers rapidly [71,72,73,74,75] so that the human operator never has to be exposed to the pathogen or risk the loss of life.

The potential for aptamers to couple to the human complement lysis cascade and general immune system for passive immunity has been explored by both the author [76,77,78,79] and the sadly deceased Nobel laureate Kary Mullis to fight bacterial pathogens [80] and cancer cells [77,78] as well. Kary Mullis’ former company Altermune, LLC, which utilized aptamers conjugated to alpha-gal epitope to couple to 1% circulating anti-alpha gal antibodies was acquired for its “alphamer” technology which is currently under advanced development at Centauri Therapeutics in the UK. Thus, there is real tangible hope for inexpensive aptamer-based passive immunity against infectious disease and cancers versus the more expensive humanized monoclonal antibody therapies.

One final, albeit rather exotic, possibility for necessary aptamer-based passive immunity lies in the fact that NASA acknowledged aptamers and SELEX as being a potentially key technology to protect astronauts on future space missions, if they should encounter live extraterrestrial microbes that are pathogenic to humans [81]. It is also worth noting that much of the robotic automated SELEX research and engineering development is microfluidic in nature [74,75,82] and would allow NASA to carry miniature automated SELEX devices into space for emergency medical countermeasures (i.e., artificial passive immunity).

## 4. Discussion and Conclusions

It has been three decades since the pioneers Tuerk, Gold, Ellington and Szostak [1,2] first published on aptamers and SELEX technology. But to this day, aptamers are still struggling to become adopted as either diagnostic reagents or pharmaceuticals, with only the anti-VEGF aptamer Macugen^®^ having received FDA approval for treatment of wet age-related macular degeneration thus far. In part, this struggle was predictable because antibodies are so entrenched as both diagnostic reagents and pharmaceuticals with major companies having made huge financial investments in their success. So instead of trying to compete directly against such overwhelming odds and potential bias, the aptamer community might be better served to adopt a cleverer strategy to identify and exploit areas in which specific aptamer properties, such as size and nucleic acid composition, make aptamers the more attractive alternative to antibodies. A few of these potential niche applications have been defined and supported by examples in this review. For example, aptamers can sometimes fill a void in which no commercial antibodies exist such as for detection of *Cyclospora* or liver flukes. Aptamers are also more easily manipulated by incorporation of exotic or unnatural bases in their binding sites during chemical synthesis of DNA or RNA versus antibodies to discriminate very similar targets. Aptamers can also utilize their smaller size and tissue penetration to enhance histochemical staining or SERS signal intensity. Aptamers can also be produced entirely in vitro, thus sparing host animals toxicity or possible death when the target antigen could be lethal. In addition, the SELEX method can be engineered into small automated or robotic microfluidic devices to provide on demand passive immunity in otherwise impossible environments such as the void of outer space. Finally, aptamers are much less expensive to develop and produce versus humanized monoclonal antibodies for passive immunity. All of these aptamer strengths or advantages can be exploited to help push aptamers further into niche applications within the diagnostic and pharmaceutical markets.

## Data Availability

Data sharing not applicable.
